# Portable and integrated microfluidic flow control system using off-the-shelf components towards organs-on-chip applications

**DOI:** 10.1007/s10544-023-00657-z

**Published:** 2023-06-02

**Authors:** Haoyu Zhu, Gürhan Özkayar, Joost Lötters, Marcel Tichem, Murali Krishna Ghatkesar

**Affiliations:** 1 Department of Precision and Microsystems Engineering, Delft University of Technology, Mekelweg 2, Zuid-Holland 2628CD Delft, The Netherlands; 2 Bronkhorst High-Tech BV, Nijverheidsstraat 1A, Ruurlo, 7261 AK Gelderland The Netherlands; 3 Faculty of Electrical Engineering, Mathematics and Computer Science, Integrated Devices and Systems, University of Twente, Drienerlolaan 5, Enschede, 7522 NB Overijssel The Netherlands

**Keywords:** Organ-on-a-chip, Fluid handling system, Portability, Fluid flow control, Flow control scheme, System design, System integration

## Abstract

Organ-on-a-chip (OoC) devices require the precise control of various media. This is mostly done using several fluid control components, which are much larger than the typical OoC device and connected through fluidic tubing, i.e., the fluidic system is not integrated, which inhibits the system’s portability. Here, we explore the limits of fluidic system integration using off-the-shelf fluidic control components. A flow control configuration is proposed that uses a vacuum to generate a fluctuation-free flow and minimizes the number of components used in the system. 3D printing is used to fabricate a custom-designed platform box for mounting the chosen smallest footprint components. It provides flexibility in arranging the various components to create experiment-specific systems. A demonstrator system is realized for lung-on-a-chip experiments. The 3D-printed platform box is 290 mm long, 240 mm wide and 37 mm tall. After integrating all the components, it weighs 4.8 kg. The system comprises of a switch valve, flow and pressure controllers, and a vacuum pump to control the diverse media flows. The system generates liquid flow rates ranging from 1.5 $\upmu$Lmin$^{-1}$ to 68 $\upmu$Lmin$^{-1}$ in the cell chambers, and a cyclic vacuum of 280 mbar below atmospheric pressure with 0.5 Hz frequency in the side channels to induce mechanical strain on the cells-substrate. The components are modular for easy exchange. The battery operated platform box can be mounted on either upright or inverted microscopes and fits in a standard incubator. Overall, it is shown that a compact integrated and portable fluidic system for OoC experiments can be constructed using off-the-shelf components. For further down-scaling, the fluidic control components, like the pump, switch valves, and flow controllers, require significant miniaturization while having a wide flow rate range with high resolution.

**Supplementary Information:**

The online version contains supplementary material available at 10.1007/s10544-023-00657-z.

## Introduction

Organ-on-a-chip (OoC) devices mimic organ functions on a micro-chip. The OoC technology is becoming a vital branch of *in vitro* biomedical analysis Convery and Gadegaard (2019). The technology promises to significantly reduce animal testing and bring down the costs of drug development Joshi (2016). OoC devices emulate the physiological environment Sosa-Hernández et al. (2018), manipulate cells Porkka-Heiskanen (2013), and precisely control biophysical and biochemical parameters Aziz et al. (2017).

OoC devices are made out of glass, silicon, or polymers, or a combination of them. The devices can also have on-chip active functions like valves, pumps, stretching membranes, etc. OoC experiments need stable and fluctuation-free fluid control, with a wide range of flow rates, typically between 0.1 $\upmu$L min$^{-1}$  and 500 $\upmu$L min$^{-1}$ , within the allowed shear stress on cells Byun et al. (2014), Piergiovanni et al. (2021), Zhu (2020). The hydrodynamics of the flow rate and flow profile induces mechanobiological response in cells Huber et al. (2018), Dessalles et al. (2021). Flow rates can modulate cell morphology, gene expression patterns, protein secretion, cell-cell adhesion and cell-matrix adhesion, thus influencing cellular behavior, for example, cancer progression and metastasis Shemesh et al. (2015). For most OoC experiments, cells are cultured inside the chip for a long time (days to weeks) and perturbed by chemical stimuli. Cells are optically monitored and various parameters (pH, oxygen level, fluorescence etc.) are recorded Wikswo et al. (2013). Multiple OoC devices are also used to collect statistics and include control experiments Probst et al. (2018).

**Fig. 1:**
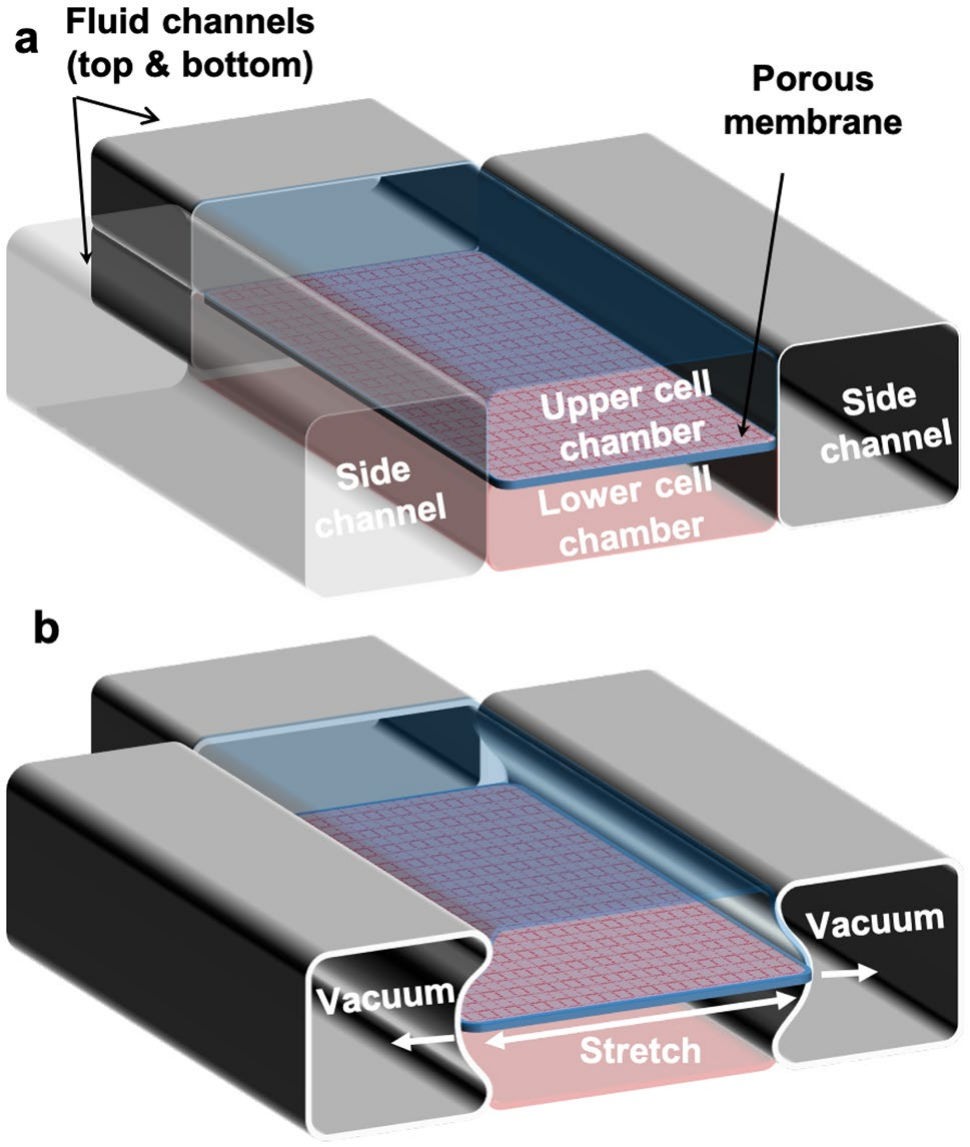
**a** Schematic of a lung-on-a-chip device with two cell chambers in the middle and two side channels. A porous horizontal membrane separates the cell chamber on top from the bottom. A thin vertical membrane separates the cell chambers from two side channels. The device is made out of Polydimethylsiloxane (PDMS) material Huh et al. (2010). **b** Vacuum in the side channels stretches the porous membrane. The chip emulates the breathing motion of the alveolus with epithelial cells grown on one side of the porous membrane and endothelial cells on the other. The upper channel allows liquid or gas flow depending on the application, while the lower channel allows only liquid flow

Among many OoC devices developed so far, lung-on-a-chip is a complex device to implement with flow control components. It needs control on liquids, air, and vacuum to perform experiments and operate the system Huh et al. (2010). A common design consists of two cell chambers on top of each other, separated by a membrane in the middle. Two additional side channels to the cell chambers are used to apply cyclic vacuum. The vacuum in the side channels stretches the middle membrane, thus mimicking lung function on the chip (see Fig. 1). Gut-on-a-chip uses a similar chip configuration Kim et al. (2012). Other simpler OoC devices such as kidney-on-a-chip use a similar chip configuration, but occasionally with a single flow channel Jang and Suh (2010). Therefore, a flow control system implemented for lung-on-a-chip can also be utilized for gut-on-a-chip or simpler OoC devices.

There is a continuous effort on fabricating various types of microfluidic chips demonstrating many OoC functionalities Sosa-Hernández et al. (2018), Yang et al. (2017), Lee and Cho (2016). There is also an effort to integrate multiple sensors like pH, temperature, photodetector, etc., inside the OoC chip Zhu et al. (2021). For the execution of experiments on these devices, many bulky peripheral fluid control components like fluid pumping sources, flow controllers, and switch valves are used. The level of miniaturisation and integration of these components is low. Therefore, OoC test setups are usually confined to a fixed location without portability. In a typical setup, it is inconvenient to move the OoC chip between different analytical instruments without disconnecting from peripheral fluid control components, leading to change in the experimental conditions.

Some of the integrated flow control systems reported are listed here. Li et al. developed a smartphone-controlled microfluidic handling system integrated with a compact pneumatic system and elastomeric chip with valves (2014). A pressure difference was used to drive a fluctuation-free flow. However, no flow controller was integrated in their system. Ma et al. developed a portable microfluidic platform for a rapid detection of pathogens Ma et al. (2019). The platform had temperature control and was integrated with a smartphone for real-time detection of parameters. However, the flow control was based on an ON/OFF mechanism without a proportional control. A few studies also showed progress on the miniaturization of peripheral components for OoC applications. For example, the multi-sensor integrated OoC platform was developed by Zhang et al. (2017). Their platform could test multiple chips and integrate multiple bioelectrochemical sensors on a large-scale flow control breadboard. However, the pump and the valve controller were not integrated. A multi-chamber OoC platform by Vollertsen et al. (2020) for real-time *in-situ* monitoring of many parallel OoC experiments did not address the integration of peripheral components. Some commercial OoC companies like Emulate, Inc. also do not address the integration of peripheral components either Instruments and Accessories (2021). A high-throughput organ-on-a-chip platform developed by Azizgolshani et al. (2021) integrates micropumps and real-time sensors. However, the system still requires an external bulky pressure source and controllers that are not integrated yet, hence lacking the portability. Cantoni et al. (2021) have integrated two commercial micropumps and temperature control for a two-channel microfluidic chip on a portable platform. Piezoelectric micropumps (without dampers) generate varying flow fluctuations depending on the chosen flow rate, therefore not suitable for cells in OoC applications. Furthermore, in their configuration, for every inlet fluid, a separate pump is needed, increasing the complexity.

In this paper, we propose a flow control configuration using a vacuum pump to minimize the number of components used and obtain a fluctuation-free flow Özkayar et al. (2022). With this configuration, we analysed the possibilities and limitations of using off-the-shelf components to create an integrated and portable flow control system for a complex OoC application. We do that by designing, building, and characterizing a flow control system for a lung-on-a-chip application. Compared to the existing flow control systems used in OoC applications, the novel aspects of our platform are the following: 1) it is reconfigurable for many OoC applications (e.g. lung, gut, kidney, tissue etc.), 2) it works on a battery, and uses a vacuum pump integrated on the platform, thus removing the need to hook the system to the walls of the laboratory during the experiment, 3) it is compact enough to move between different analytical instruments without disconnecting the OoC chip from the flow control system.

**Fig. 2:**
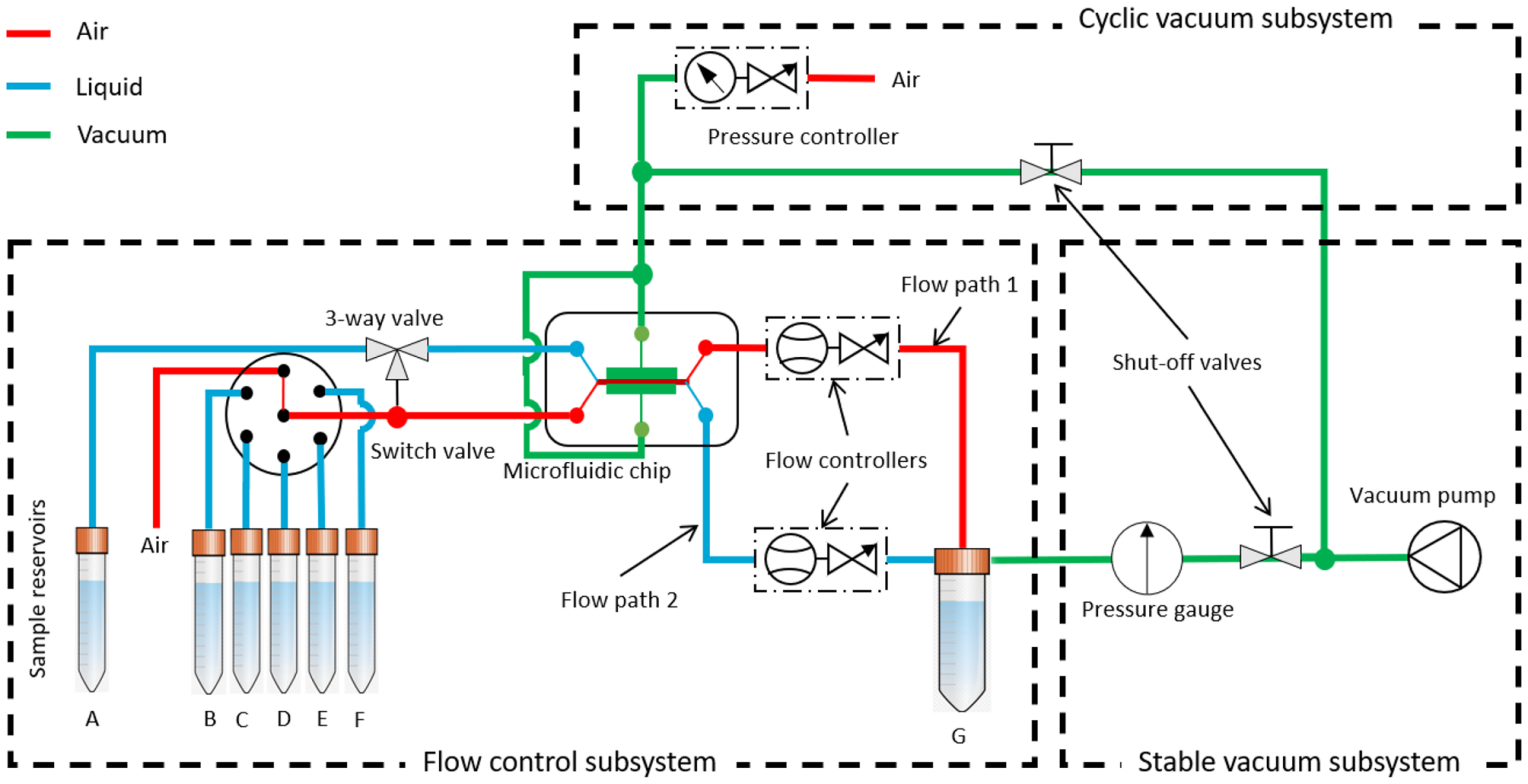
Schematic diagram of the fluid handling system, consisting of three subsystems: 1) a stable vacuum subsystem, 2) a flow control subsystem, and 3) a cyclic vacuum subsystem. 1) The stable vacuum subsystem generates the necessary under-pressure to drive fluids in the system. The shut-off valves separate the vacuum pump from different subsystems. 2) The flow control subsystem contains sample reservoir tubes A to F kept at ambient pressure and a waste reservoir tube G at vacuum. The vacuum in tube G enables the aspiration of fluid samples from different reservoirs to the waste reservoir while passing through the switch valve, the microfluidic chip, and flow controllers. For two cell chambers (Fig. 1), there are two fluid flow paths in the chip. The fluid flow rate in both flow paths is controlled separately by controlling the valve in the flow controllers (compatible with both gas and liquid). The fluid is routed in both channels by a switch valve and a 3-way valve. 3) The cyclic vacuum subsystem is used to control the stretching of the membrane separating two cell chambers. The shut-off valve to the flow control subsystem is closed, and the shut-off valve to the cyclic vacuum subsystem is opened. The pressure controller provides the desired frequency cycles of the vacuum

## System design

This section describes the system requirements, the connection scheme for fluidics, fluid control design, and a 3D-printed platform to optimally integrate the components and make the entire system portable.

### System requirements

To meet the requirements of lung-on-a-chip, gut-on-a-chip and simpler OoC applications mentioned in Section 1, the general and specific requirements are listed below.

#### General requirements

The system should be able to (1) perform experiments on at least three types of OoCs separately lung-on-a-chip, gut-on-a-chip and kidney-on-a-chip, (2) flow fluid without disconnecting any tubing during the entire experiment, (3) control the flow rate for each channel independently, (4) integrate all peripheral flow control components on a single platform, (5) mount on an upright and inverted optical microscope to monitor cells, (6) accommodate in an incubator without disconnecting any tubing or electrical power cables, and (7) operate in a user friendly manner.

#### Specific requirements

The system should be able to (1) cover a liquid flow rate range of 0.3 $\upmu$Lmin$^{-1}$ - 50 $\upmu$Lmin$^{-1}$ with the stability of $\geqslant 1\%$ Relative Standard Deviation (RSD) Huh et al. (2010), Benam et al. (2016), Hancock and Elabbasi (2017), (2) generate a fluctuation-free fluid flow, (3) have cyclic vacuum (to stretch the membrane in Lung-on-a-chip) is $\ge$ 0.2 Hz, (4) cover a vacuum range from $-$280 mbar to $-$80 mbar (5 % to 15 % strain in a lung-on-a-chip device Huh et al. (2010)), (5) be able to handle both air and liquid, and (6) work on a battery for at least 72 hours on full load. Note that negative values of pressure indicates below atmosphere.

### Connection scheme

#### Fluid handling system scheme

The proposed fluid handling system consists of (1) a stable vacuum subsystem, (2) a flow control subsystem, and (3) a cyclic vacuum subsystem (Fig. 2). (1) In the stable vacuum subsystem, a vacuum pump generates the necessary pressure difference for the fluid flow in the entire system. Two shut-off valves separate the vacuum pump load either from the flow control subsystem or the cyclic subsystem. (2) In the flow control subsystem, fluid flow in the cell chambers of the OoC is controlled. A vacuum in the waste reservoir generates a pressure difference between the inlet (sample reservoirs) and the outlet (waste reservoir). This pressure difference drives fluid from the sample reservoirs through the cell chambers to the wast reservoir. The flow rate in each cell chamber is controlled by an independent flow controller (compatible for both gas and liquid) in their respective flow paths. A desired fluid in the cell chamber is selected by a fluid selector switch valve. The same or different fluids in both cell chambers are selected by a 3-way valve. (3) In the cyclic vacuum subsystem, the vacuum pump is connected to the side chambers of the OoC to stretch the membrane separating the cell chambers. The pressure-flow controller connected to the vacuum on the one side and the ambient air on the other side controls the vacuum at a programmed frequency to mimic the breathing function in a lung-on-a-chip experiment.

The names, brands and model keys of the off-the-shelf components used in the system are listed in Table 1. The components with smallest footprint and a range compatible with OoC applications were chosen. More detailed information is in the supplementary information Fig. S1, S2 and S3 respectively.

**Table 1:** List of the functional components used in the system

Name	Brand	Model Key
Vacuum pump	SURGEFLO	-
Vacuum gauge	Festo	VAM-63-V1/0-R1/4-EN
Shut-off valve	SMC	VX214AGA
Shut-off valve	Festo	HE-2-QS-6
Switch valve	IDEX	MHP7970-500-4
3-way valve	SMC	VDW-250-1-G-2-01F-A-Q
Flow controller	Bronkhorst	ML120V21-BAD-CC-K-S-DA-A0V
Pressure controller	Bronkhorst	IQP-600C-1K5A-AAD-00-V-A

#### Control electronics scheme

**Fig. 3:**
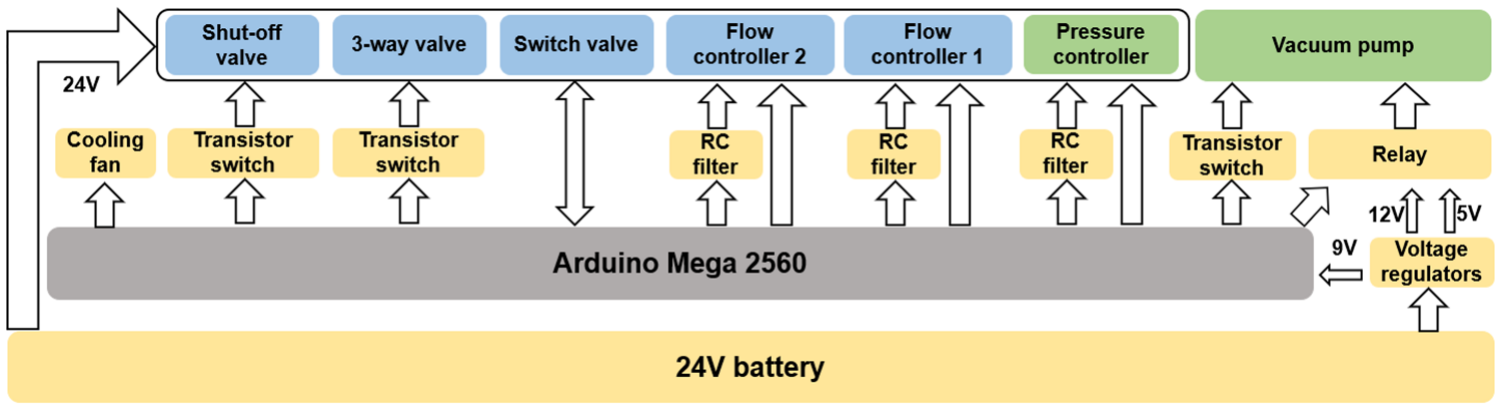
An Arduino-based electronic control scheme for flow control is shown. The yellow blocks represent the intermediate electronic components that interface with the electronics of the flow control components, voltage regulators, and power resources. The blue blocks represent the electronics of the flow control components. The green blocks represent the pressure control components. The RC filters convert the pulse-width modulated output signal from Arduino to analog setpoints for the controllers

An Arduino Mega 2560 microcontroller (see Fig. 3) was used to control all the fluid flow control components listed in Table 1. The flow control components, micro-controller and different electronic components were powered by 24 V, 9 V, 5 V and 12 V, respectively. All these different voltages were derived from a 24 V battery pack using voltage regulators. The vacuum pump was operated at either 12 V or 5 V, switched by a relay, depending on the need to either rapidly obtain high vacuum or to maintain the vacuum at reduced power consumption, low noise and low vibration. A fan was used to cool down the regulators during the operation of cyclic vacuum. The PCB with the microcontroller and all the control electronic components was placed at the bottom layer of the platform (see supplementary information Figs. S4 and S5).

### 3D-printed platform and assembly

A 3D-printed box is used as a platform to integrate all the components of the flow control system, including the battery that powers the entire system. 3D-printing gives the design freedom to adapt the platform for a particular microscope, incubator, or flow control configuration. Also, it has the advantage of flexible design in terms of accommodating diverse components and their arrangement, low-cost, and rapid prototyping.

**Fig. 4:**
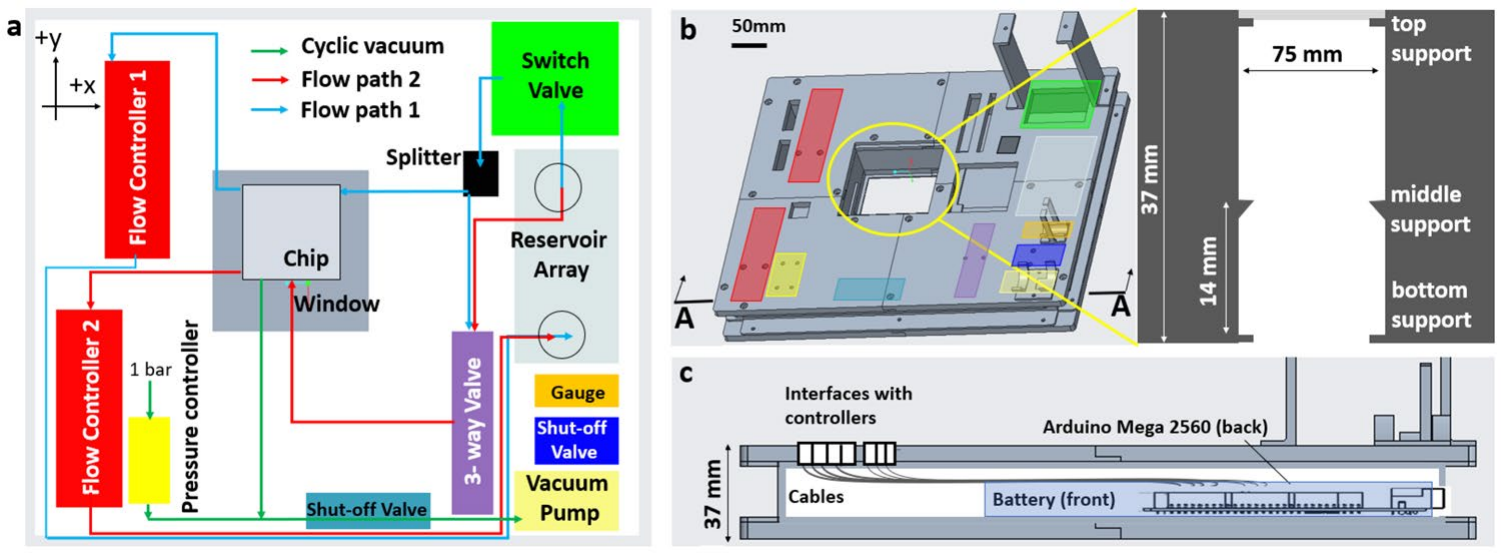
The platform design architecture. **a** The physical layout of off-the-shelf components and the fluid flow path. **b** A CAD model of the box that holds all the control electronics. The component layout on top of the box is shown. The box is 290 mm long, 240 mm wide and 37 mm tall. Three frames on top of the box secured the switch valve, vacuum gauge, and vacuum pump. Zoom-in picture shows the cross-section of a 75 mm $\times$ 75 mm cut-out in the box. The organ chip can be mounted on the top, bottom, or at 14 mm height inside the cut-out region, suitable for inverted and upright microscopes. **c** The cross-section of the box with securing frames on the top-right. The electronic components: Arduino, control PCB, and battery were placed inside the box

Figure 4 shows the integrated platform design layout. The flow control components (Table 1) are placed on the top of the box and control electronics inside the box. The component layout on top of the box is shown in Fig. 4a, and the CAD picture of the same in Fig. 4b. The electronic printed circuit boards and batteries placed inside the box are shown in Fig. 4c. The box has an opening of 75mm x 75mm that fits the size of more than one of the most organ chips. Support structures in the opening along the box thickness hold the organ chips at the desired height (shown as a zoom-in side view in Fig. 4b), suitable for either upright or inverted optical microscopes. The box is printed in several separate parts made from polylactic acid (PLA) and joined together. Such a design allows to print only a sub-part of the entire platform box whenever changes are needed. For the CAD model of separate sub-parts of the box, please refer to supplementary information Figs. S11 to S14. Additional information on the printed sub-parts and assembly are given in the supplementary information The printed material PLA may not be suitable for autoclave. However, it is compatible with sterilizing liquids like 70 % Ethanol and 25 % Hydrogen peroxide.

**Fig. 5:**
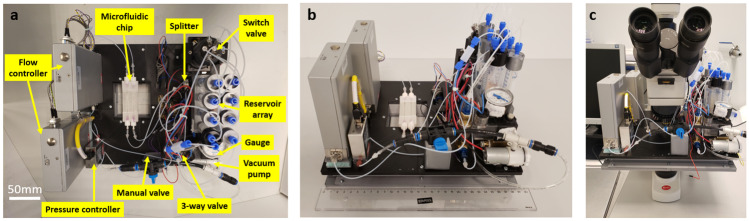
The assembled setup of a battery-operated, programmable, portable, and integrated OoC platform. **a** Top view of the platform with functional components labeled. **b** Isometric-view of the platform whose dimensions are 290 mm long, 240 mm wide and 220 mm tall (37 mm box + components on top), with a total weight of 4.8 kg. All fluidic components were placed on the box. The control electronic components and the batteries were inside the box. **c** The platform was mounted on an upright microscope (Motic, BA310Met-T). The microfluidic (OoC) chip was placed on top of the cut-out window in the box Fig. (4b). The 37 mm tall platform box fits on the stage stably, with the weight of the components evenly distributed. It was possible to manually move the x-y stage and focus inside the microfluidic channels with 5x, 10x, 20x, and 40x objectives

## Results

### Assembled system

The integrated and portable OoC system is shown in Fig. 5. It is 290 mm long, 240 mm wide and 220 mm tall (37 mm box + components on top), with a weight of 4.8 kg. It can be easily carried by a single person and fits in an incubator. A top view and isometric view of the platform with assembled components layout are shown in Fig. 5a, b respectively. To avoid cross-talk between flow controllers, a vibration isolation foam support between one of the flow controllers and the base was used. The platform can also be placed stably on a microscope stage, as shown in Fig. 5c. It can be placed on an upright or an inverted optical microscope.

### A protocol example for a lung-on-a-chip experiment

In order to illustrate the sequential functioning of the platform for an OoC application, main flow control steps in a protocol for a lung-on-a-chip experiment is given in Table 2 Huh et al. (2010), Benam et al. (2016), Hancock and Elabbasi (2017). The schematic diagrams of the steps and their correlative timing diagrams indicating the activation of different components with time can be found in the supplementary information figures, Figs. S6-S10.

The lung-on-a-chip has two cell chambers, with air flowing through the top chamber (red color) and liquid flowing through the bottom chamber (blue color) (Fig 2). The vacuum is applied in the side channel (green) to stretch the membrane and mimic breathing function in the chip. Before starting the protocol, vacuum is generated in the waste reservoir by switching the vacuum pump ON at 12V, open the shut-off valve connected to the flow control. After 10min the vacuum is switched to pump at 5V, to maintain vacuum in the waste reservoir.

The actions taken to implement Lung-on-a-chip protocol given in Table 2 are as following. *Step 1*: Sterilization. An externally sterilized OoC chip is mounted on the setup. Ethanol is used to sterilize the OoC channels and chambers. The switch valve is connected to the conditioning media, e.g., reservoir tube B. The 3-way valve is connected to the switch valve, which closes the connection to tube A. The valves in both flow controllers are fully open to allow the desired flow rate for the conditioning process (see supplementary information Fig. S6). *Step 2*: Seeding epithelial cells (air-blood barrier). Epithelial cells are flown and allowed to deposit on the top side of the membrane (top cell chamber) through flow path 1, e.g., reservoir tube C. The switch valve is connected to tube C. The 3-way valve is connected to tube A. The flow controller in flow path 2 is closed by setting the flow rate to zero, and the flow controller in flow path 1 is open to flow liquid from the top cell chamber (see supplementary information Fig. S7). *Step 3*: Seeding endothelial cells (walls of blood capillary). Endothelial cells are flown and allowed to deposit on the bottom side of the membrane (bottom cell chamber) through flow path 2, e.g., reservoir tube D. The switch valve is connected to tube D. The 3-way valve is connected to the switch valve. The flow controller in flow path 1 is closed by setting the flow rate almost to zero, and the flow controller in flow path 2 is open to flow liquid from the bottom cell chamber (see supplementary information Fig. S8). *Step 4*: Cell culture. Culture media is flown in the top and bottom cell chambers by appropriately choosing the switch valve and adjusting the flow controllers in both flow paths. The three-way valve is connected to tube A to separate both flow paths (see supplementary information Fig. S9). *Step 5*: Mimic lung breathing. The top cell chamber is connected to air by connecting the switch valve to the air channel. The 3-way valve is connected to tube A, flowing liquid through the bottom cell chamber. Note that flow controllers in flow paths 1 and 2 can control air and liquid flow. The shut-off valve in the cyclic vacuum subsystem is open, connecting to a vacuum pump, and the pressure controller is operated in cycles of the desired frequency. The vacuum pump is powered at 5 V, and the shut-off valve in the stable vacuum system is open (see supplementary information Fig. S10). All these steps were automated by programming in the Arduino microcontroller.

From various protocols published on lung-on-a-chip devices Huh et al. (2010), Benam et al. (2016), Hancock and Elabbasi (2017), the most critical steps from the perspective of the fluid type and flow rate range are shown in Table 2. Please refer to the cited articles for the complete protocols. Table 2 illustrates that the developed system can achieve the performance requirements of lung-on-a-chip application. These parameters also satisfy the requirements of gut-on-a-chip de Haan et al. (2019), liver-on-a-chip Carraro et al. (2008) and certain combination of organs-on-a-chip Zhang et al. (2017) requirements.

**Table 2:** Main flow control steps in a protocol for a lung-on-a-chip experiment Huh et al. (2010), Benam et al. (2016), Hancock and Elabbasi (2017)

Step	Process	Type of fluid	Flow rate
1	Conditioning of the channels and cell chamber	Ethanol, DI water	50 $\upmu$Lmin$^{-1}$
2	Epithelial cell seeding on one side of the membrane in the cell chamber	Culture medium with cells	Static or 1.5 $\upmu$Lmin$^{-1}$
3	Endothelial cell seeding on another side of the membrane in the cell chamber	Culture medium with cells	Static or 1.5 $\upmu$Lmin$^{-1}$
4	Cell culture	Culture medium	1.5-50 $\upmu$Lmin$^{-1}$
5	Emulating breathing by cyclic stretching of the membrane.	Air, Vacuum	0.15-0.25 Hz, 10-15 % mechanical strain

### Flow control and vacuum generation

The maximum vacuum generated in the waste reservoir is $-$610 mbar when the shut-off valve in the stable vacuum subsystem (Fig. 2) is closed. This vacuum in the waste reservoir is gradually lost over time as the liquid flows through the flow paths 1 and 2. The loss in vacuum does not influence the fluid flow rate in both paths because the flow controllers adjust the flow rate in real time. The program is designed to open the shut-off valve in the stable vacuum subsystem once every 10 minutes to maintain the necessary vacuum in the reservoir.

#### Flow rate control with applied voltage

The flow rate was converted to  $\upmu$Lmin$^{-1}$ from g/h using Eq. (1) for water.

1 $$\begin{aligned} Q_V = \frac{1000 \times Q_M}{60 \times \rho }, \end{aligned}$$

where $Q_M$ represents the mass flow rate and $Q_V$ the volumetric flow rate. $\rho$ represents the density of the fluid. The density of DI water used in the test was 1 g cm^–3^.

The input voltage applied to the flow controllers was converted into a number that was processed by the Arduino microcontroller as given by Eq. (2).

2 $$\begin{aligned} V_{in} = \frac{5 \times N}{255}, \end{aligned}$$

where $V_{in}$ is the input voltage applied to flow controllers, while N represents the number that should be set in the Arduino program.

#### Settling time, resolution, stability tests

**Fig. 6:**
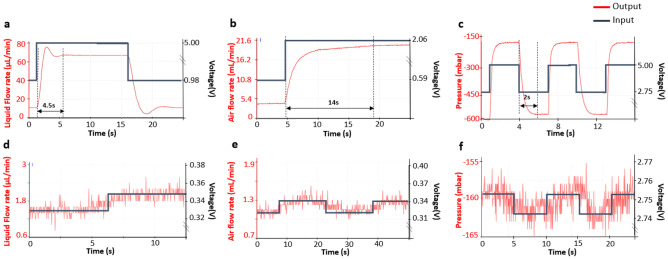
Responses of the flow controllers for liquid flow, airflow, and vacuum level. Figures **a** and **d** show a settling time of 4.5 s and a resolution of 0.6 $\upmu$L min$^{-1}$ respectively for the flow controller with liquid flow. Figures **b** and **e** show a settling time of 14 s and a resolution of 0.2 mL min$^{-1}$ respectively for the flow controller with airflow. Figures **c** and **f** show a settling time of 2 s a resolution of 3 mbar respectively for the flow controller used to generate the desired vacuum. The black lines represent the applied voltage levels (shown on the right side of the y-axes) to the controllers, while the red lines are the system responses (shown on the left side of the y-axes). The dashed lines represent that the settling times are defined as the result reaches within 2 % of the desired stable values. These values are suitable for a lung-on-a-chip experiment parameters described in Table 2. The noise seen in Fig 6d-f is due to 8-bit ADC. It can be improved by using higher bit ADC or averaging the data. According to the manufacturer, the accuracy of the mass flow controller is ${0.167\,\mathrm{\upmu \text {L}\ \text {min}^{-1}}}$ for water and ${0.13\,\mathrm{\upmu \text {L}\ \text {min}^{-1}}}$ for air, and of the pressure controller is ${0.075\,\mathrm{\text {bar}}}$ for air

The response characteristics of the flow and pressure controllers is shown in Fig. 6. The characteristics were tuned by adjusting the PID (proportional, integral and differential) parameters of the respective controller. The settling time is defined as the amount of time the controllers takes to reach the 2 % of the desired valve for a step input. The resolution is defined as the minimum value of a parameter that can be resolved. In the present setup, it is defined by the 8-bit analog-digital-converter (ADC) of the Arduino controller. The stability is defined as the relative standard deviation of a parameter over a measured time. The settling time, measurement resolution and stability of the flow controllers for water flow, air flow and vacuum level are shown in Fig. 6.

**Fig. 7:**
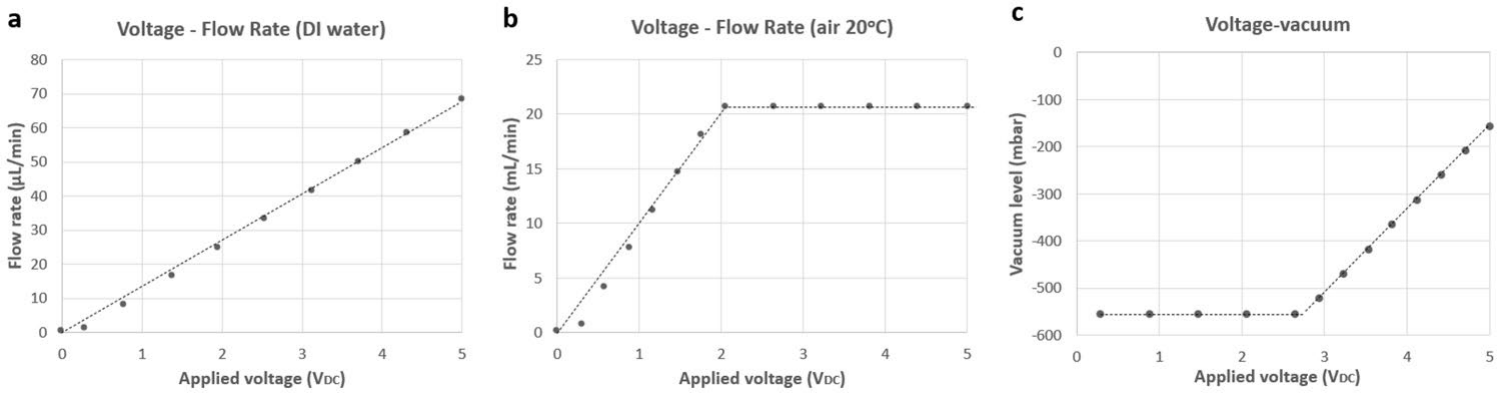
System calibration curves for the normally-closed flow controller and pressure controller. **a** Flow rate calibration for liquid flowing through the flow controller with applied voltage. **b** Flow rate calibration for air flowing through the controller. The data for **a** and **b** were collected by connecting the flow controller between the liquid reservoir and the waste reservoir connected to the vacuum pump. **c** Vacuum calibration with applied voltage for air flowing through the pressure controller. The system shows good reproducibility

For water flow, the flow controller had a response time of 4.5 s (Fig. 6a), resolution of 0.6 $\upmu$L min^–1^ (Fig. 6d) and a stability of 0.58 % measured over 60 minutes. The quantitative relationship between the volume flow of DI water at room temperature and the applied voltage is $Q_V = 13.57 \times U$, with the unit of  $\upmu$Lmin$^{-1}$. This equation is derived from Fig 7a.

For air flow, the flow controller had a response time of 14 s (Fig. 6b), a resolution of 0.2 mL min^–1^ (Fig. 6e) and a stability of 0.56 % measured over 60 minutes. The quantitative relationship between air at room temperature and the applied voltage is $Q_V = 10.54 \times U$ while $0.33 \le U \le 1.96$ and $Q_V = 20.66$ while $1.96 \le U \le 5$. The unit for this equation is mL min^–1^. These equations are derived from Fig 7b.

For the vacuum level in the side chambers, the pressure controller had a response time of 2 s, a resolution of 3 mbar and a stability of 0.16 %. The quantitative relationship between side chamber vacuum degree and applied voltage is as follows $Q_V = -556$ while $0 \le U \le 2.75$ and $Q_V = 177.73 \times U - 1045.3$ while $2.75 \le U \le 5$. The unit for the equation is mbar. These equations are derived from Fig 7c.

Applying these results in the context of a lung-on-a-chip experiment (Table 2), the response time obtained for liquid and gas flow controllers are fast enough for many experiments with cells. However, the flow rates are close to the limits of the controller. Furthermore, based on the chip design and the application, other OoC devices need flow rates as low as 0.3 $\upmu$L min$^{-1}$ or as high as 500 $\upmu$L min$^{-1}$.

### Power consumption

The power consumption of the vacuum pump is 3.12 W when operated at 12 V, and 0.55 W at 5 V. The vibration and noise from the vacuum pump were greatly reduced when operated at 5 V. The maximum power consumption of the switch valve, shut-off valve, 3-way valve, flow controllers and pressure controller were 0.5 W, 4.5 W, 3 W, 3 W and 1.5 W respectively. Powered by a 24 V, 2000 mAh battery, the working duration of the system varied from 3 to 96 hours based on different applications for a total power consumption varying between 0.5 W and 18.5 W. The battery is rechargeable and, if needed. easily exchangeable.

### Display of fluid flow and vacuum

**Fig. 8:**
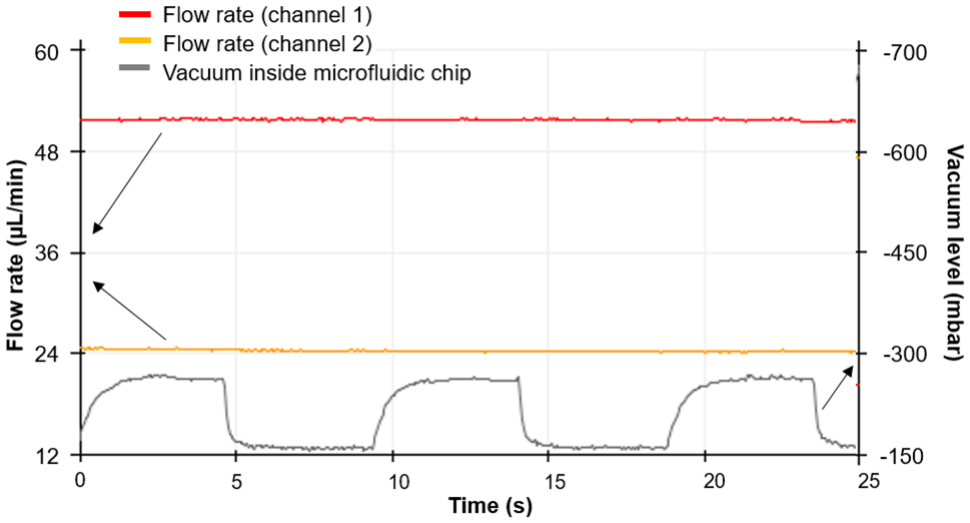
Flow rate/vacuum display. The y-axis on the left shows different flow rates for fluid flowing through flow path 1 and flow path 2. The y-axis on the right shows the (cyclic) vacuum level. The x-axis represents time. In this particular example, the liquid flow rate in flow path 1 was 51 $\upmu$Lmin$^{-1}$ while the liquid flow rate in path 2 was 24 $\upmu$Lmin$^{-1}$. Vacuum level in the cyclic vacuum subsystem was from $-$157 mbar to $-$280 mbar with a period of 0.1 Hz, desired values in lung-on-a-chip applications. The arrows show the relevant y-axis for the data

The system was connected to a computer with an Arduino program and a display to monitor the flow rate and vacuum level in the same window, as shown in Fig. 8. The y-axis on the left shows the flow rate of either liquid or air flow through the flow controllers in flow path 1 and flow path 2 (see Fig. 2). The y-axis on the right shows the vacuum level. Since a window can show a fixed number of samples (500), if the sampling interval is 50 ms, the duration for each window will be $500 \times 0.05$, which is 25 s as shown in Fig. 8.

## Discussion

Table 3 shows the overall characteristics of the system. Most of the general and specific design requirements have been met. The fluid carrying components (see Fig 2): the switch valve, 3-way valve, and flow controllers are compatible with biological liquids De Haan and Pim (2019). However, the system reached a minimum liquid flow rate of 1.5 $\upmu$L min$^{-1}$ and a vacuum of $-$157 mbar, instead of the required 0.3 $\upmu$L min$^{-1}$ and $-$80 mbar, respectively. Consequently, shear stress on the cells cannot be reduced below the lowest flow rate, and the membrane separating the top and bottom cell chambers cannot be stretched to the desired length. According to the flow controller manufacturer, the minimum liquid flow rate the controller can measure is 0.8 $\upmu$L min$^{-1}$. Regular auto-zeroing of the flow controller could enable it to reach the minimum value of the flow controller. The system will still not reach the required 0.3 $\upmu$L min$^{-1}$. Being modular, replacing the flow controller with another type that operates in the suitable range will solve the problem Zeng et al. (2021), Lötters and Joost (2012). Leakages in the connectors did not allow the side channels to reach the maximum vacuum but is enough to produce the required strain in the membrane.

In the flow channel paths, no bubbles were observed. It is important to note that all the connections should be well-sealed as the system uses a vacuum to generate the flow. Any leakage at the connectors will lead to bubbles in the flow and potentially damage the cells. The usage of bubble traps before the OoC chip is safe and removes any undesired bubbles if generated. Though the vibrations due to the vacuum pump are small even at 12V, the consequence on cells should be tested, and appropriate vibration isolation for OoC chip should be considered. Typically, the polymer-based chips should dampen the undesired vibrations, if any, before reaching cell chambers. The flow control components and the 3D printed box can be sterilized, enabling the system to be placed inside an incubator. However, this has yet to be tested.

The system was designed for a maximum of three OoC devices, for a standard glass slide (25 mm $\times$ 75 mm) each. Enabling the system for more than three OoC chips will involve redesign. Modularity of the design enables to easily adapt to the chosen flow control components and schemes.

**Table 3:** Characterized performance of the portable OoC platform

**Category**	**Parameter**	**Performance**
Application	Channel types	2 (flow)+1 (vacuum)
	Fluid type	Liquid, Gas and Vacuum
	Liquid flow range	1.5 - 68 $$\upmu$$Lmin$$^{-1}$$$$^{a}$$
	Gas flow range	1.3 - 20.7 mL min^-1^$$^{b}$$
	Vacuum range	$-$157 - $-$556 mbar
	Flow profile	Fluctuation-free
Architecture	Platform size (L $\times$ W $\times$ H)	29 cm $\times$ 24 cm $\times$ 22 cm
	Weight	4.8 kg
	Cost	€ 5600
Control	Function	Process control
	Controllability	Linear
		Liquid$^a$: 0.8 $\upmu$Lmin$^{-1}$
	Resolution	Gas$^b$: 0.2 mL min^-1^
		Vacuum: 3 mbar
		Liquid$^a$: $\ge$ 0.58 % RSD*
	Flow stability	Gas$^b$: $\ge$ 0.56 % RSD*
		Vacuum: $\ge$ 0.16 % RSD*
		Liquid$^a$: 4.5 s
	Settling time	Gas$^b$: 14 s
		Vacuum: 2 s
Battery	Power consumption	Minimum: 0.5 W
		Maximum: 18.5 W
	Battery worktime	Minimum: 3 h
		Maximum: 96 h

^a^DI water is the tested liquid. The value may change if the liquid changes

^b^The fluid is the air at a temperature of 20$^{\circ }$C

## Conclusions and future work

A portable and integrated microfluidic handling platform for OoC that need mechanical stimuli has been designed, fabricated, and characterized. A combination of small footprint off-the-shelf components that operates on a battery and a 3D printed platform enabled to achieve the requirements. The use of vacuum produced a fluctuation-free pumping and reduced the number of pumps needed for the setup. The 3D-printed platform allows customer-specific system designs. The materials chosen for the platform can be sterilized. To avoid condensation on electronic systems, pre-heating the platform before putting it inside the incubator can be considered. In terms of performance, most of the parameters are in the typical OoC working range. The liquid flow lower range should be a minimum of 0.3 $\upmu$L min$^{-1}$ and a maximum of 500 $\upmu$L min$^{-1}$, where we could reach 1.5 $\upmu$L min$^{-1}$ and 68 $\upmu$L min$^{-1}$, respectively. The limitation was mainly from the specifications of the chosen off-the-shelf flow controller.

Our future work includes: a) testing the platform inside an incubator to check if the electronic circuitry survives the high humidity, b) testing cell viability and lung-on-a-chip protocol, c) to develop a low-power temperature Elhassan (2023) and humidity control module on the platform for the OoC chips, d) to miniaturize flow controllers that cover the full range of OoC applications Zeng et al. (2021), Lötters et al. (2012), Groenesteijn et al. (2016), and e) to miniaturize the switch valve Gunda et al. (2020). Finally, up-scaling flow control for many tens of OoCs will improve the statistics on the data obtained and additional control experiments in the same conditions, thus making the platform useful for personalized medicine.

## Supplementary Information

Below is the link to the electronic supplementary material.

Supplementary file1 (PDF 2.13 MB)
