# Supplementary material for: Portable and integrated microfluidic flow control system using off-the-shelf components towards organs-on-chip applications
Source: Biomed Microdevices. 2023 Jun 2;25(2):19. doi: 10.1007/s10544-023-00657-z (PMC10238329; doi:10.1007/s10544-023-00657-z)
Supplement: Supplementary file 1 — Supplementary file1 (PDF 2.13 MB) [file 10544_2023_657_MOESM1_ESM.pdf]

Supplementary information: Portable and Integrated Microfluidic Flow Control System Using Off-the-shelf Components Towards Organs-on-chip Applications

Haoyu Zhu[1†], Gürhan Özkayar[1], Joost Lötters[1,2,3], Marcel Tichem[1] and Murali Krishna Ghatkesar[1*]

[1*]Department of Precision and Microsystems Engineering, Delft University of Technology, Mekelweg 5, Delft, 2628CD, Zuid-Holland, The Netherlands.
[2]Bronkhorst High-Tech BV, Nijverheidsstraat 1A, Ruurlo, 7261 AK, Gelderland, Country.
[3]Faculty of Electrical Engineering, Mathematics and Computer Science, Integrated Devices and Systems, University of Twente, Drienerlolaan 5, Enschede, 7522 NB, Overijssel, The Netherlands.

*Corresponding author(s). E-mail(s): M.K.Ghatkesar@tudelft.nl;
†Present address: Department of Mathematics and Computer Science, Eindhoven University of Technology, Eindhoven, The Netherlands

1 Flow control design

1.1 Stable vacuum subsystem

The stable vacuum subsystem generates the necessary pressure difference for fluid flow in the system.

The sequence of operation is given below. (1) Open the normally-closed shut-off valve (24 V) to the flow control subsystem. (2) Switch-on the vacuum pump (12 V) for 5 s. (3) Close the shut-off valve to seal the vacuum inside the waste reservoir. Applying 12 V to the vacuum pump ensures a maximum

vacuum of 610 mbar below atmospheric pressure in the reservoir commensurate with the performance of the vacuum pump used. The vacuum in the reservoir can be maintained by opening the shut-off valve and keeping the pump running at 5 V instead of 12 V. This low voltage significantly minimizes the pump vibrations, noise and power consumption. Periodically (every 10 min for 5 s) operating the pump at a higher voltage compensates for the vacuum loss with time.

1.2 Flow control subsystem

The flow control subsystem steers the fluid and controls the flow rate through the OoC. The flow rate in the flow control subsystem as described in Fig. 2 is given as

$$Q = \frac{\Delta P_{Const}}{R_{Tube} + R_{Switch} + R_{Splitter} + R_{Chip} + R_{Cont}}, \tag{1}$$

where ΔP_{const} is pressure difference generated in the waste reservoir created by the vacuum pump, R_{Tube}, R_{Switch}, $R_{Splitter}$ and R_{Cont} are hydraulic resistances of the connecting tubes, switch valve, 3-way valve splitter and tubing inside the flow controller respectively. Two Cori-FLOW mass flow controllers (R_{Cont}) from Bronkhorst High-Tech B.V. controlled the fluid flow. They use the Coriolis principle to measure the flow rate. The desired flow rate is programmable in the microcontroller connected to the flow controllers.

A switch valve and a 3-way valve controlled multiple fluids through the OoC chip (Fig. 2). The IDEX switch valve used had six fluid inlet channels and one fluid outlet channel. Each channel was connected to different fluids. By applying 4-line BCD control signals, the stepper motor in the valve rotated and connected to the desired fluid position. The microcontroller reads and controls valves' status (ON or OFF). The 3-way valve ensures the same liquid in both cell chambers, e.g., DI water or ethanol, to initialize the chips. After initialization, the valve separated flow in the top and bottom cell chambers. Flow path 1 allows gas or liquids from tubes B to F and flow path 2 allows a constant liquid flow from tube A. The flow path 1 is connected to the epithelial cell chamber and flow path 2 to the endothelial cell chamber for lung-on-a-chip application.

1.3 Cyclic vacuum subsystem

Stretching of the membrane separating both cell chambers by cyclic vacuum (≈ 0.2 Hz) in the side chambers emulated the breathing function of a lung-on-a-chip. As shown in Fig. 2, the side channels can switch connections between vacuum pump or atmosphere with a T-junction. The flow path to the atmosphere has a flow controller controlling the amount of vacuum in the side channels. The amount of vacuum needed, the stretching frequency, and the waveform shape is programmable in the microcontroller. The pump can create a vacuum of at least −500 mbar in the side chambers.

Name	Brand	Model Key	Link of Manual/Website	Page	Unit Price	Total Price	Way of Obtaining
Vacuum Pump	SURGEFLO	-	https://nl.aliexpress.com/item/33006096807.html?spm=a2g0s.9042311.0.0.4e144c4dlqjenH	-	1.39	1.39	Purchase from Aliexpress
Shut-off Valve	SMC	VX214AGA	https://docs.rs-online.com/ad6b/0900766b813f122e.pdf	12-13	45.52	45.52	Supported by PME Department
Vacuum Gauge	Festo	VAM-63-V1/0-R1/4-EN	https://www.festo.com/us/en/a/download-document/datasheet/537811/	-	26.36	26.36	Supported by PME Department
Manual Valve	Festo	HE-2-QS-6	https://docs.rs-online.com/94e1/0900766b816a12cc.pdf	-	18.34	18.34	Supported by PME Department
Switch Valve	IDEX	MHP7970-500-4	https://www.idex-hs.com/store/pub/media/productattachments/files/downloads/File-1469551785.pdf	-	2075.11	2075.11	Purchase form Inacom
Three-way Valve	SMC	VDW-250-1-G-2-01F-A-Q	https://www.smcworld.com/discon/en/oldpdf/vdw-old-e.pdf	402-406	22.21	22.21	Purchase form RS online
Flow Controller	Bronkhorst	ML120V21-BAD-CC-K-S-DA-A0V	https://www.bronkhorst.com/getmedia/84f1ce1f-9a10-4007-b89d-873031f9ad9f/917097-Manual-mini-CORI-FLOW-ML120.pdf	-	1516.95	3033.9	Supported by Bronkhorst
Pressure Controller	Bronkhorst	IQP-600C-1K5A-AAD-00-V-A	https://www.bronkhorst.com/getmedia/aef32966-8264-45d8-8478-b40df22290ab/917045-Manual-IQ-FLOW.pdf	-	233.3	233.3	Supported by Bronkhorst
						5456.13	

Fig. S1 List of flow control components used in the platform

Name	Quantity	Parameter	Way of Obtaining
Reservoir	8	10ml	Supported by PME Department
Leak tight cap	1	-	Supported by PME Department
PTFE tubing	-	1/16" X 1/32"	Supported by PME Department
PU tubing	-	4mm X 2.5mm	Supported by PME Department
PU tubing	-	6mm X 4mm	Supported by PME Department
Plastic thread fitting and Ferrule	10	1/4"-28 to 1/16" OD	Supported by PME Department
Plastic fitting	1	1/4"-28 to 2.5mm ID	Supported by PME Department
Metal thread fitting and Ferrule	7	1/4"-28 to 1/16" OD	Purchase from Inacom
Barbed adapter	7	1/4"-28 to 1/16" OD	Supported by PME Department
Syringe needle	4	0.25mm	Supported by PME Department
Screw	34	M3 X 12	Supported by PME Department
Screw	4	M3 X 16	Supported by PME Department
Screw	2	M4 X 12	Supported by PME Department
Screw	4	M5 X 12	Supported by PME Department
Screw	2	UNC 4-40	Purchase from RS online
Nut	38	M3	Supported by PME Department

Fig. S2 List of flow control connections used in the platform

Name	Brand	Quantity	Link of Manual/Website	Parameter	Unit Price	Total Price	Way of Obtaining
Arduino Mega 2560	Arduino	1	https://docs.rs-online.com/9ab3/0900766b80e8ba22.pdf	-	46.94	46.94	Purchase from RS online
Battery 1	HENSENS	1	https://www.123accu.nl/Soundcast-20S-1P-accu-24V-2000-mAh-123accu-huismerk-i27294.html?mkwid=sGugb8Byg_dc%7Cpcrid%7C171663098801%7Cpkw%7C%7Cpmt%7C%7Cslid%7C%7Cprid%7CASO00611_4894128104582&pgrid=40095430284&ptaid=pla-297471473451&gclid=EAIaIQobChMI9LD_lYKx6QIVlrt3Ch0RTQapEAQYBSABEgKxpPD_BwE	24VDC	47.5	47.5	Purchase from 123accu
Battery 2	TIANQIU	1	https://tianqiu.en.made-in-china.com/product/KvtmsGnHaYkb/China-6f22-9V-Tianqiu-Super-Heavy-Duty-Carbon-Dry-Battery.html	9VDC	1.1	1.1	Purchase from Aliexpress
Cooling fan	ebm-papst	1	https://docs.rs-online.com/d403/0900766b816d6d50.pdf	5V,0.4W	24.9	24.9	Purchase from RS online
Resistor	-	1	-	220Ω	-	-	Supported by PME Department
Resistor	-	2	-	4.7kΩ	-	-	Supported by PME Department
Resistor	-	3	-	8.5kΩ	-	-	Supported by PME Department
Transistor	STMicroelectronics	3	https://docs.rs-online.com/d061/0900766b8135f82f.pdf	80V,3A	2.69	2.69	Purchase from RS online
Voltage regulator	STMicroelectronics	1	https://docs.rs-online.com/9aaa/0900766b814b2420.pdf	12V,1.5A	0.417	0.417	Purchase from RS online
Voltage regulator	STMicroelectronics	1	https://docs.rs-online.com/61d4/0900766b813d3857.pdf	5V,1.5A	0.559	0.559	Purchase from RS online
Capacitor	EPCOS	3	https://nl.rs-online.com/web/p/polyester-film-capacitors/0334221/	100nF	0.15	0.45	Purchase from RS online
General purpose PCB	ADAFRUIT INDUSTRIES	1	https://docs.rs-online.com/82cc/0900766b8153435e.pdf	-	8.05	8.05	Purchase from RS online
Internet cable	Omron	1	https://docs.rs-online.com/504e/0900766b815165a2.pdf	-	11.4	11.4	Purchase from RS online
RJ45 connector	MH Connectors	1	https://docs.rs-online.com/9abe/0900766b813030b5.pdf	-	1.26	1.26	Purchase from RS online
9 pin D sub connector Male	TE Connectivity	2	https://docs.rs-online.com/6181/0900766b804c485c.pdf	-	4.302	4.302	Purchase from RS online
9 pin D sub connector Female	RS PRO	4	https://docs.rs-online.com/20e6/0900766b815860ec.pdf	-	0.929	0.929	Purchase from RS online
SPST Switch	Arcolectric	2	https://docs.rs-online.com/af05/0900766b808a97c8.pdf	-	2.2	2.2	Purchase from RS online
Relay	TONGLING	1	https://www.generationrobots.com/media/JQC-3FF-v1.pdf	5VDC	0.61	0.61	Purchase from Aliexpress
						153.307	

Fig. S3 List of electronic connections used in the platform

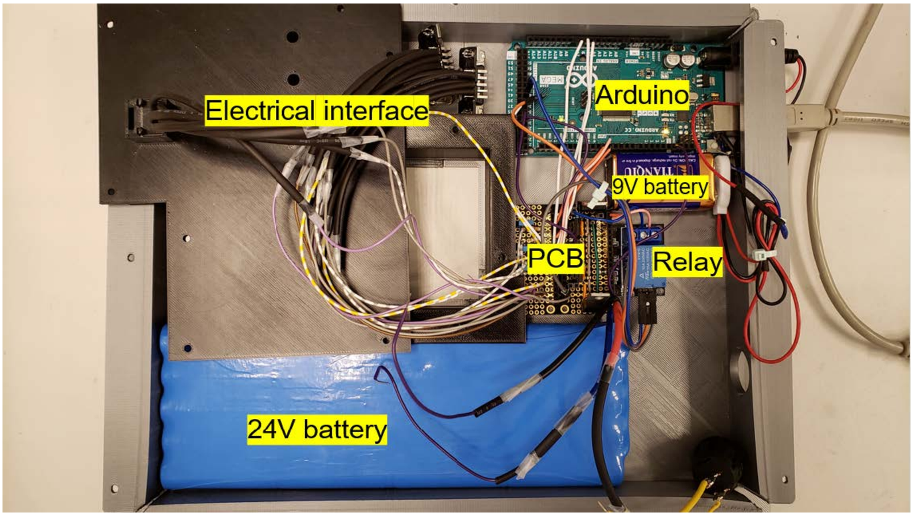

Fig. S4 Photo of electronics placed in the base of platform.

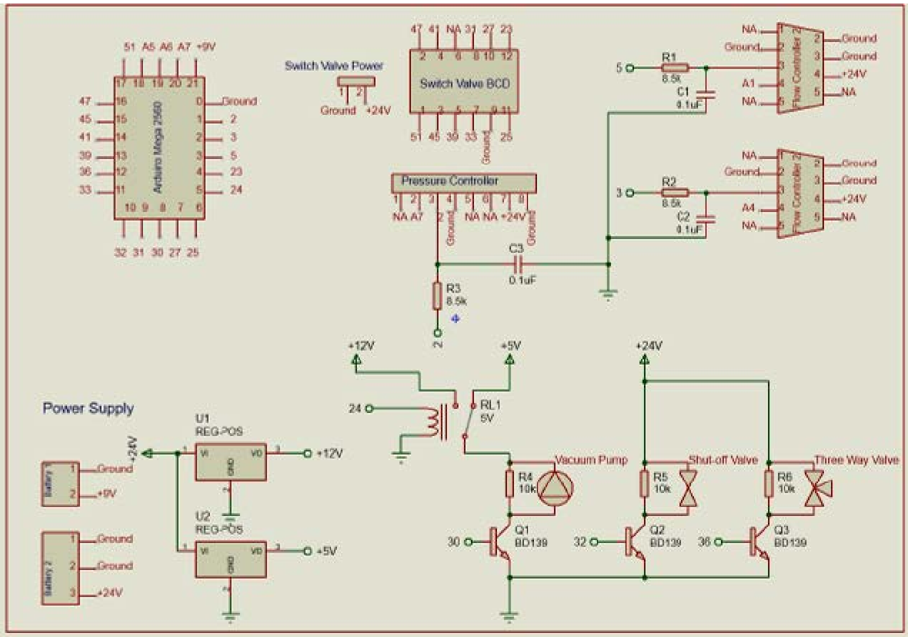

Fig. S5 Electronic diagram of the system including an simplified Arduino diagram with all the used connections at the upper-left corner.

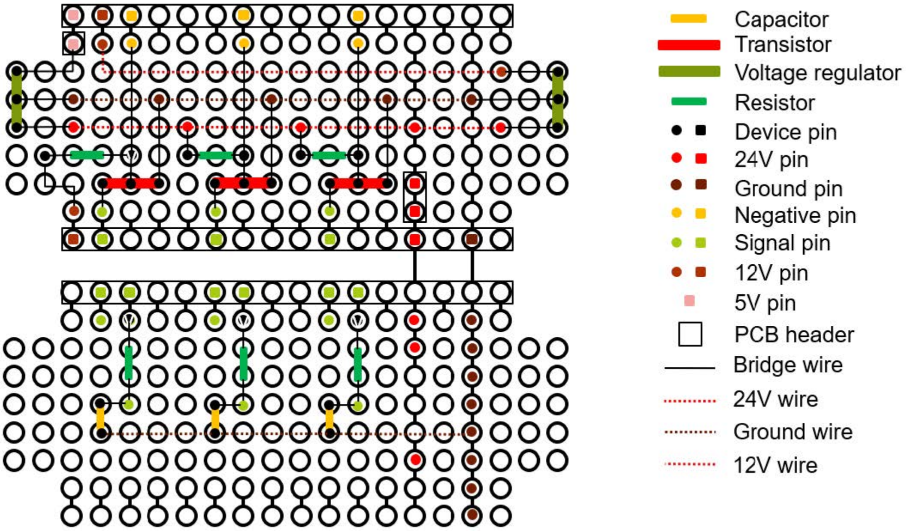

Fig. S6 Layout of general purpose PCB. The dash lines represent wires in the back side while other devices are placed at the front side.

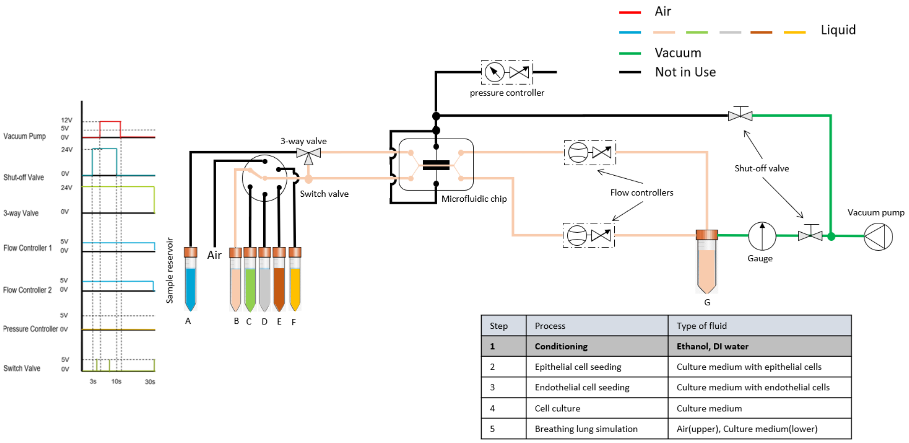

Step	Process	Type of fluid
**1**	**Conditioning**	**Ethanol, DI water**
2	Epithelial cell seeding	Culture medium with epithelial cells
3	Endothelial cell seeding	Culture medium with endothelial cells
4	Cell culture	Culture medium
5	Breathing lung simulation	Air(upper), Culture medium(lower)

Fig. S7 Protocol of Lung-On-Chip experiments - conditioning process. The flow rate controlled by the Flow controller 1 and 2 are set to the largest.

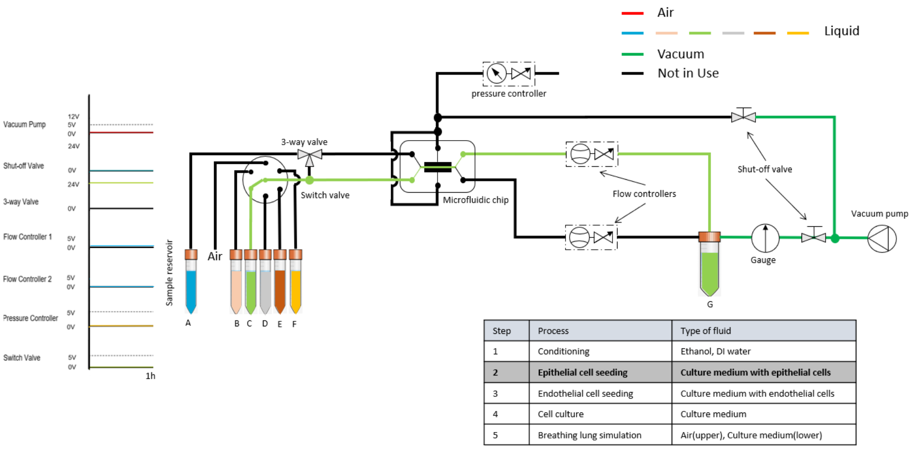

Step	Process	Type of fluid
1	Conditioning	Ethanol, DI water
**2**	**Epithelial cell seeding**	**Culture medium with epithelial cells**
3	Endothelial cell seeding	Culture medium with endothelial cells
4	Cell culture	Culture medium
5	Breathing lung simulation	Air(upper), Culture medium(lower)

Fig. S8 Protocol of Lung-On-Chip experiments - epithelial cell seeding process. The flow rate controlled by the Flow controller 2 is low but not equal to zero.

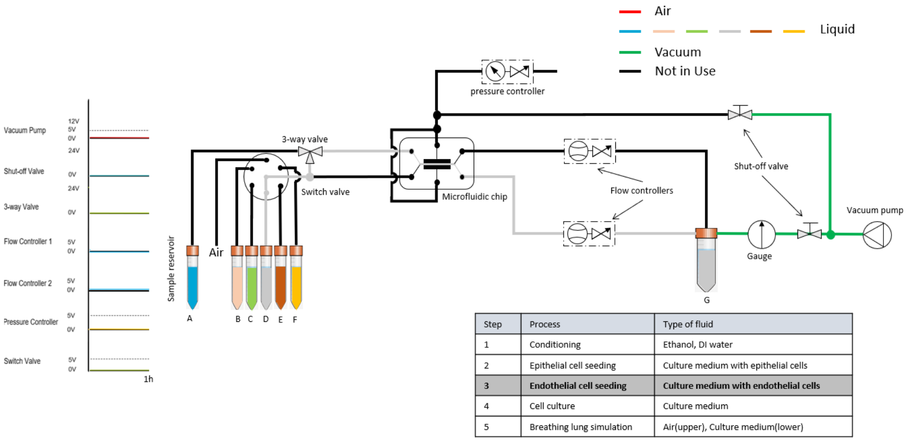

Step	Process	Type of fluid
1	Conditioning	Ethanol, DI water
2	Epithelial cell seeding	Culture medium with epithelial cells
**3**	**Endothelial cell seeding**	**Culture medium with endothelial cells**
4	Cell culture	Culture medium
5	Breathing lung simulation	Air(upper), Culture medium(lower)

Fig. S9 Protocol of Lung-On-Chip experiments - endothelial cell seeding process. The flow rate controlled by the Flow controller 1 is low but not equal to zero.

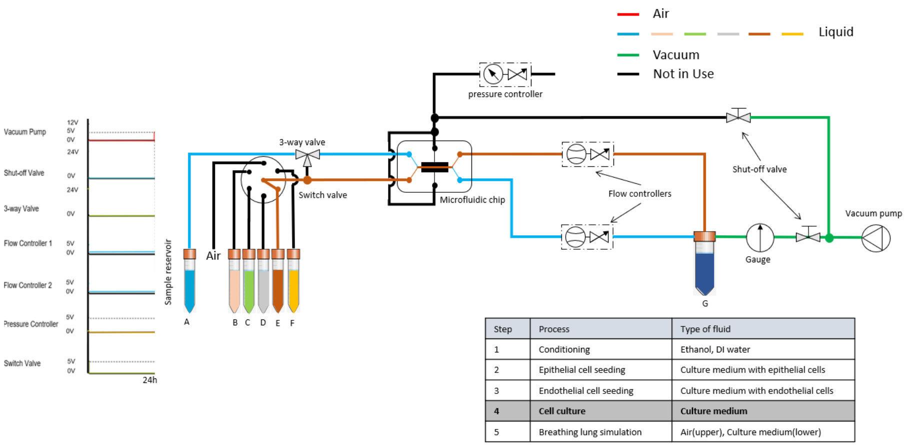

Step	Process	Type of fluid
1	Conditioning	Ethanol, DI water
2	Epithelial cell seeding	Culture medium with epithelial cells
3	Endothelial cell seeding	Culture medium with endothelial cells
**4**	**Cell culture**	**Culture medium**
5	Breathing lung simulation	Air(upper), Culture medium(lower)

Fig. S10 Protocol of Lung-On-Chip experiments - cell culture process. The flow rate controlled by the Flow controller 1 and 2 are suitable for cell culture.

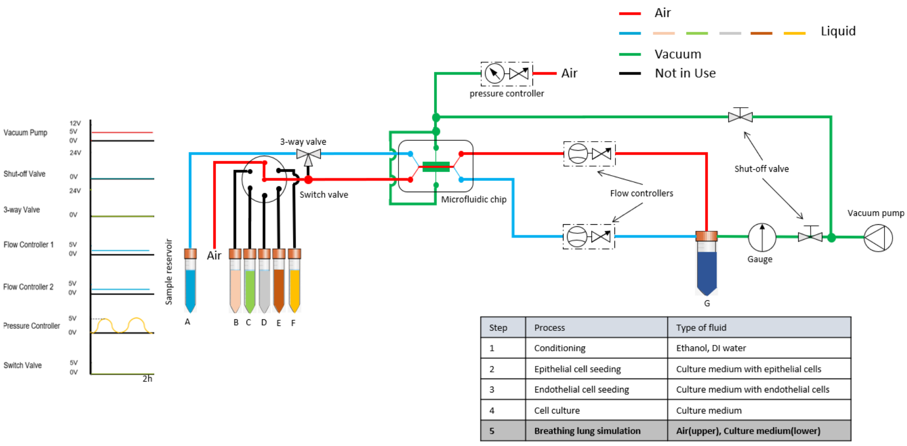

Step	Process	Type of fluid
1	Conditioning	Ethanol, DI water
2	Epithelial cell seeding	Culture medium with epithelial cells
3	Endothelial cell seeding	Culture medium with endothelial cells
4	Cell culture	Culture medium
**5**	**Breathing lung simulation**	**Air(upper), Culture medium(lower)**

Fig. S11 Protocol of Lung-On-Chip experiments - breathing lung simulation. The flow rate controlled by the Flow controller 1 and 2 are selected based on the user's application.

<table>
<tr><td rowspan="4">Four pieces used to form a bottom plate. The pieces are fixed with each other by step structures with M3 through holes. Other holes on plate are used to fix with supports.</td><td>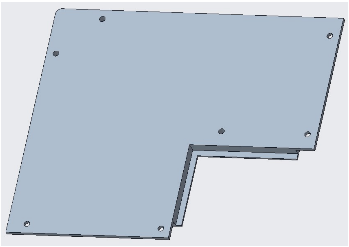</td></tr>
<tr><td>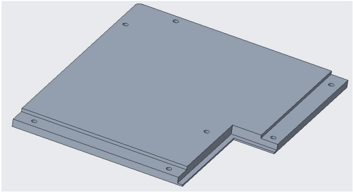</td></tr>
<tr><td>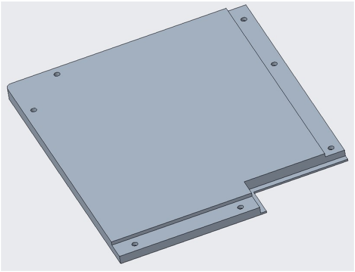</td></tr>
<tr><td>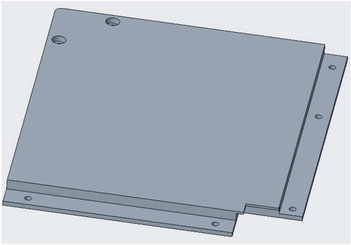</td></tr>
</table>

Fig. S12 Sub-parts used to assemble the bottom plate of the box.

<table>
<tr><td rowspan="4">Four pieces used to form a top plate. The pieces are fixed with each other by step structures with M3 through holes. Other holes on plate are used to fix with supports and other fluidic components such as pumps, vlaves and their frames. The square holes are used to connect fluidic components with electronic interfaces by D-sub, RJ45, Molex 12pin, and general PCB headers.</td><td>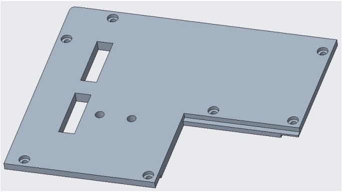</td></tr>
<tr><td>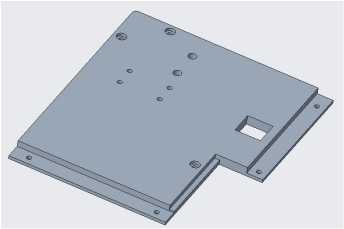</td></tr>
<tr><td>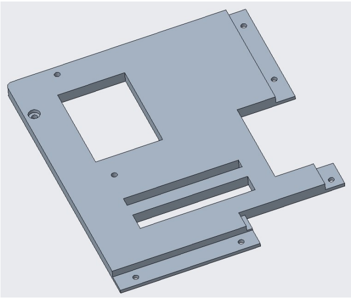</td></tr>
<tr><td>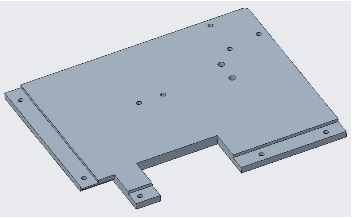</td></tr>
</table>

Fig. S13 Sub-parts used to assemble the top plate of the box.

<table>
<tr><td rowspan="4">Supports used to separate top and bottom plates. The third picture shows the middle support which is also used to form a window for chip accommodation. The square hole in first picture is used to connect Arduino microcontroller to outside laptop and make room for battery connection and its switch. The circle hole is used to accommodate the switch for the entire system.</td><td>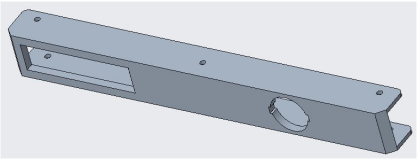</td></tr>
<tr><td>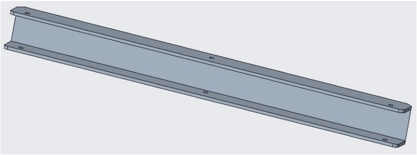</td></tr>
<tr><td>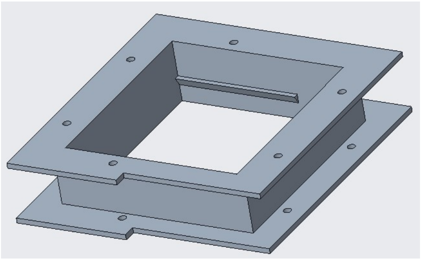</td></tr>
<tr><td>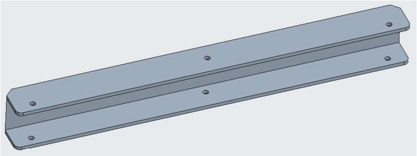</td></tr>
</table>

Fig. S14 Sub-parts used to separate and support the bottom and top plates.

Support frame for fixing the gauge. The curve part is used to fit the splitter connected to gauge and the structure is fixed by double-side tape in curvature.	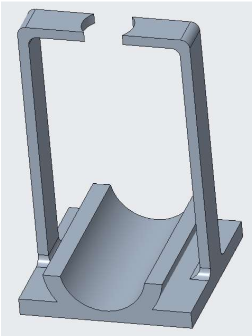
The frame used to fix switch valve. The valve is vertically placed and two UNC 4-40 screws are used to fix the switch valve through the holes on top of the frame.	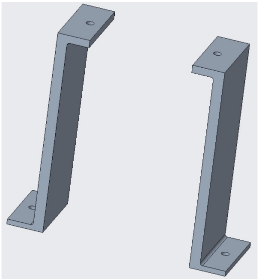
The frame used to fix vacuum pump. The pump has a cylinder structure thus can be fixed by double-side types in the curvature part. Holes are used to fix the frame on top of the top plate.	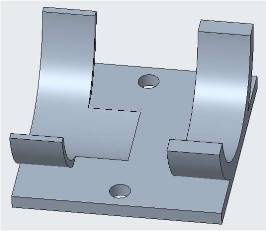
The support used to place the manual valve at higher location to decrease the stress in vacuum tubes. The structure are all connected by double-side types.	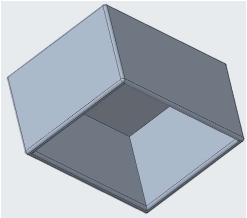

Fig. S15 Sub-parts used to fix the flow control components.
